# Pharmacologic inhibition of bone resorption prevents cancer-induced osteolysis but enhances soft tissue metastasis in a mouse model of osteolytic breast cancer

**DOI:** 10.3892/ijo.2014.2468

**Published:** 2014-05-27

**Authors:** IRENE ZINONOS, KE-WANG LUO, AGATHA LABRINIDIS, VASILIOS LIAPIS, SHELLEY HAY, VASILIOS PANAGOPOULOS, MARK DENICHILO, CHUN-HAY KO, GRACE GAR-LEE YUE, CLARA BIK-SAN LAU, WENDY INGMAN, VLADIMIR PONOMAREV, GERALD J. ATKINS, DAVID M. FINDLAY, ANDREW C.W. ZANNETTINO, ANDREAS EVDOKIOU

**Affiliations:** 1 Discipline of Surgery, Breast Cancer Research Unit, Basil Hetzel Institute and Centre for Personalised Cancer Medicine, University of Adelaide, Adelaide, Australia; 2 Institute of Chinese Medicine and State Key Laboratory of Phytochemistry and Plant Resources in West China, The Chinese University of Hong Kong, Hong Kong, P.R. China; 3 Department of Radiology, Memorial Sloan-Kettering Cancer Center, New York, NY, USA; 4 Discipline of Orthopaedics and Trauma, University of Adelaide, Adelaide, Australia; 5 Myeloma Research Laboratory, School of Medical Sciences, Faculty of Health Science and Centre for Personalized Cancer Medicine, University of Adelaide, Adelaide, Australia; 6 School of Medicine at the Basil Hetzel Institute, The Queen Elizabeth Hospital, Woodville and the Robinson Institute, University of Adelaide, Adelaide, Australia

**Keywords:** osteoprotegerin, bone metastasis, bone resorption, antiresorptive agents, bisphosphonates

## Abstract

Osteoprotegerin (OPG) is a secreted member of the TNF receptor superfamily, which binds to the receptor activator of nuclear factor κB ligand (RANKL) and inhibits osteoclast activity and bone resorption. Systemic administration of recombinant OPG was previously shown to inhibit tumor growth in bone and to prevent cancer-induced osteolysis. In this study, we examined the effect of OPG, when produced locally by breast cancer cells located within bone, using a mouse model of osteolytic breast cancer. MDA-MB-231-TXSA breast cancer cells, tagged with a luciferase reporter gene construct and engineered to overexpress full-length human OPG, were transplanted directly into the tibial marrow cavity of nude mice. Overexpression of OPG by breast cancer cells protected the bone from breast cancer-induced osteolysis and diminished intra-osseous tumor growth but had no effect on extra-skeletal tumor growth. This effect was associated with a significant reduction in the number of osteoclasts that lined the bone surface, resulting in a net increase in bone volume. Despite limiting breast cancer-mediated bone loss, OPG overexpression resulted in a significant increase in the incidence of pulmonary metastasis. Our results demonstrate that inhibition of osteoclastic bone resorption by OPG when secreted locally by tumors in bone may affect the behaviour of cancer cells within the bone microenvironment and their likelihood of spreading and establishing metastasis elsewhere in the body.

## Introduction

Breast cancer is the leading cause of cancer death among women worldwide, accounting for 23% of the total cancer cases and 14% of the cancer deaths in 2008 (1). Despite significant improvements in detecting and treating early breast cancer, an estimated 75–80% of patients with advanced disease develop bone metastasis (2). The pathologic complications of bone metastasis can have devastating effects and patients experience debilitating skeletal-related events (SREs), including pathological fractures, hypercalcaemia of malignancy and spinal cord compression. SREs are accompanied by severe bone pain and loss of mobility, which eventually leads to reduction of quality of life and survival (3–5).

Osteoprotegerin (OPG) is a secreted member of the TNF receptor superfamily and plays an important role in bone remodelling and osteoclastogenesis (6,7). Osteoblasts and stromal cells express receptor activator of NF-κB ligand (RANKL), which binds to its receptor RANK on pre-osteoclasts and stimulates their differentiation and maturation into functional osteoclasts (6). OPG, produced by osteoblasts and other cell types, binds to RANKL and prevents the association between RANKL and RANK, thereby inhibiting osteoclast activation and function. Mice lacking OPG exhibit severe osteoporosis (8), whereas transgenic mice overexpressing OPG develop osteopetrosis (6). Several lines of experimental evidence demonstrated that systemic administration of recombinant OPG inhibits tumor growth in bone by inhibiting osteoclast function and prevents bone loss in animal models of experimental bone metastasis (9,10). Similarly, Corey *et al* demonstrated that OPG produced locally by prostate cancer cells had similar anti-osteolytic and anti-metastatic effects (11). However, contrary to these findings, Fisher *et al* reported that local overexpression of OPG by MCF-7 breast cancer cells co-expressing parathyroid hormone-related protein enhanced tumor growth in bone and increased osteolysis (12). Moreover, there is evidence showing that high circulating levels of OPG in the serum of patients with prostate cancer appear to be predictive of increased bone metastases and increased osteolysis (13,14). Taken together these findings indicate that OPG plays a significant but perhaps context specific role in bone metastases, with evidence supporting an anti-osteoclastogenic and tumor inhibiting action, while in certain other situations it appears to stimulate osteolysis and tumor growth. These apparently conflicting observations suggest the need for additional research to delineate the role of OPG in bone malignancies.

In this study we investigated the biological effects of inhibiting bone resorption and bone remodelling on the behaviour of breast cancer cells in bone. Specifically, we examined whether OPG, when secreted locally by breast cancer cells in bone, can inhibit osteolysis and tumor growth within the bone. Our data demonstrate that overexpression of OPG by breast cancer cells diminished intraosseus tumor growth and protected the bone from breast cancer-induced osteolysis. However, despite the bone protection, OPG overexpression led to a significant increase in the incidence and severity of pulmonary metastasis. Taken together, our data demonstrate that pharmacologic inhibition of bone remodelling and bone resorption may in some cases affect the behaviour of cancer cells within the bone microenvironment and their likelihood of spreading and establishing metastasis elsewhere in the body.

## Materials and methods

### Cells and reagents

The MDA-MB-231 derivative cell line, MDA-MB-231-TXSA was kindly provided by Dr Toshiyuki Yoneda (formerly at University of Texas Health Sciences Centre, San Antonio, TX). Cells were cultured in Dulbecco’s modified Eagle’s medium (DMEM, Gibco, Cat. No. 12430-054), supplemented with 2 mM glutamine, 100 IU/ml penicillin, 160 μg/ml gentamicin, HEPES (20 mM) and 10% fetal bovine serum (Invitrogen, Cat. No. 11995-073), in a 5% CO_2_-containing humidified atmosphere. The MB-231-TXSA-TGL human breast cancer cell line has been tested and authenticated by CellBank Australia (Wentworthville, NSW, Australia) using short tandem repeat (STR) profiling (Report No. 13-163). The generation of luciferase-tagged MDA-MB-231-TXSA-TGL-p-RUF and p-OPG overexpressing human breast cancer cells were described previously (15).

### In vitro osteoclast assays

Human peripheral blood mononuclear cells (PBMCs) from healthy donors were isolated from buffy coats acquired from the Australian Red Cross Blood Service. The cells were diluted in Hank’s balanced salt solution (HBSS) and separated by gradient centrifugation with Lymphoprep (Axis Shield, Cat. No. 1114547). Isolated cells (2.5×10^5^ cells/well) were then plated in minimal essential medium (aMEM, Sigma-Aldrich, Cat. No. M4526), supplemented with 10% fetal calf serum, L-glutamine (2 mM), HEPES (20 mM), recombinant human M-CSF (25 ng/ml; Millipore, Cat. No. GF053), 1α,25(OH)_2_vitamin D3 (10 nM; Wako Industries, Cat. No. 031-14281) and dexamethasone (10 nM; Hospira, Cat. No. 483356) into osteologic slides (BD Biosciences, Cat. No. 354609), for bone resorption assays, or directly into 96-well plates for tartrate resistant acid phosphatase (TRAP) staining. The following day, media from each well was removed and replaced with fresh media supplemented with recombinant human RANKL (50 ng/ml; Millipore, Cat. No. GF091), in the presence or absence of 10% conditioned media from MDA-MB-231-TXSA-TGL-p-RUF and p-OPG-overexpressing cells. Conditioned media (CM) was replaced every 3 days. Cells were fixed on Day 7 and stained histochemically for TRAP (Sigma-Aldrich, 386-A), and TRAP+ve cells were visualized by light microscopy. To assess bone resorption, osteologic slides were stained with Von Kossa stain and resorption pits were counted using a light microscope.

### Animals

Five week old female athymic nude mice (Institute of Medical and Veterinary Services Division, Gilles Plains, SA, Australia) were acclimatized to the animal housing facility for a minimum period of 1 week prior to the commencement of experimentation. The general physical wellbeing and weight of animals were monitored continuously throughout the experiments. All mice were housed under pathogen free conditions and all experimental procedures on animals were carried out with strict adherence to the rules and guidelines for the ethical use of animals in research and were approved by the Animal Ethics Committees of the University of Adelaide and the Institute of Medical and Veterinary Science, Adelaide, SA, Australia.

### Intratibial injection model

MDA-MB-231-TXSA-TGL-p-RUF and p-OPG overexpressing human breast cancer cells were cultured in Dulbecco’s modified Eagle’s medium (DMEM), supplemented with 2 mM glutamine, 100 IU/ml penicillin, 160 μg/ml gentamicin, HEPES (20 mM) and 10% fetal bovine serum in a 5% CO_2_-containing humidified atmosphere, until they reached 70–80% confluency. Adherent cells were removed from flasks with 2 mM EDTA and resuspended in 1X PBS at 0.5×10^5^ cells/10 μl and kept on ice in an Eppendorf tube. Mice (n=10/cell line) were anaesthetised by Isoflurane (Faulding Pharmaceuticals, SA, Australia), the left tibia was cleaned with 70% ethanol and a 27-gauge needle coupled to a Hamilton syringe was inserted through the tibial plateau with the knee flexed and 0.5×10^5^ cells resuspended in 10 μl of PBS were injected into the marrow space. Tumor volume was monitored regularly and mice were imaged on a weekly basis by bioluminescence imaging (BLI) (see below). Mice were humanely sacrificed 4 weeks after cancer cell transplantation, due to high tumor load and lungs and tibiae were removed for assessment of tumor burden and bone volume, respectively.

### Preparation of blood serum and detection of OPG by ELISA

Blood was collected from all the mice at Day 18 (tail bleeds), and at the time of termination of the experiment, to determine the OPG concentration in the blood serum of the mice. Blood was collected in MiniCollect tubes (0.8 ml LH Lithium Hep Sep) and serum was separated and transferred into fresh Eppendorf tubes and stored at −80°C until assay. The concentration of OPG in blood serum collected from all animals was determined using a commercial ELISA kit as per the manufacturer’s instructions (Immunodiagnostik AG, Cat. No. KB 1011).

### Bioluminescence imaging (BLI) of tumor growth

Non-invasive, whole body imaging for assessment of tumor growth was performed once weekly using the IVIS 100 Imaging system (Xenogen, Alameda, CA). Mice were injected i.p. with 100 μl of the D-Luciferin solution at a final dose of 3 mg/20 g mouse body weight (Biosynth, Cat. No. L-82220) and then gas-anaesthetized with Isoflurane (Faulding Pharmaceuticals). Images were acquired for 0.5–30 sec (images are shown at 1 sec) from the side angle and the photon emission transmitted from mice was captured and quantitated in photons/sec/cm^2^/sr using Xenogen Living image (Igor Pro version 2.5) software.

### Micro-computed (μCT) tomography analysis

Limbs for μCT analysis were surgically resected and scanned using the SkyScan-1174 high-resolution μCT Scanner (Skyscan, Belgium). For μCT scanning, the tibiae were placed vertically in tightly fitting plastic tubes. The μCT Scanner was operated at 50 kV, 800 μA, 0.4 rotation step, 0.25 mm aluminium filter and scan resolution of 7.78 μm/pixel. The cross sections were reconstructed using a cone-beam algorithm (software NRecon, Skyscan). Files were then imported into CTAn software (Skyscan) for 3D analysis and 3D image generation. Using the 2D images obtained from the μCT scan, the growth plate was identified and 400 sections were selected starting from the growth plate/tibial interface and moving down the tibia. All images were viewed and edited using CTvol visualisation software. Histograms, representing bone volume (mm^3^), total and trabecular, were generated from tumor-bearing tibiae and compared to the contralateral non-tumor bearing tibiae. Tumor burdens, measured in mm^3^, were determined using the Skyscan software.

### Histology

Tibiae were fixed in 10% (v/v) buffered formalin (24 h at 4°C), followed by 2–4 weeks of decalcification in 0.5 M EDTA/0.5% paraformaldehyde in PBS, pH 8.0 at 4°C. Complete decalcification of the tibiae was confirmed by radiography and tibiae were then paraffin embedded. Five micron longitudinal sections were prepared and stained with H&E. Additional sections were used for osteoclast-specific tartrate-resistant acid phosphatase 5 (ACP5/TRAP) staining, following the manufacturer’s protocol (Sigma-Aldrich, 386-A). Analysis was performed on an Olympus CX41 microscope and photo-images were taken using the Nanozoomer Digital Pathology (NDP-Hamamatsu). Tumor area measured in mm^2^ was assessed using the Nanozoomer software. Lungs were also fixed in 10% (v/v) buffered formalin and were then paraffin- embedded and sectioned at 5 μm at three different levels, followed by H&E staining. Total lung area and metastatic foci area were measured in mm^2^ using the Nanozoomer software.

### Data analysis and statistics

Experiments were performed in triplicate, and data are presented as mean ± SE. All statistical analysis was performed using SigmaStat for Windows version 3.0 (Systat Software, Inc., Port Richmond, CA), using the unpaired Student’s t-test. Comparisons among groups were assessed using a one-way ANOVA test. In all cases, p<0.05 was considered statistically significant.

## Results

### OPG produced by MDA-MB-231-TXSA-p-OPG cells is biologically active

**Figure 1:**
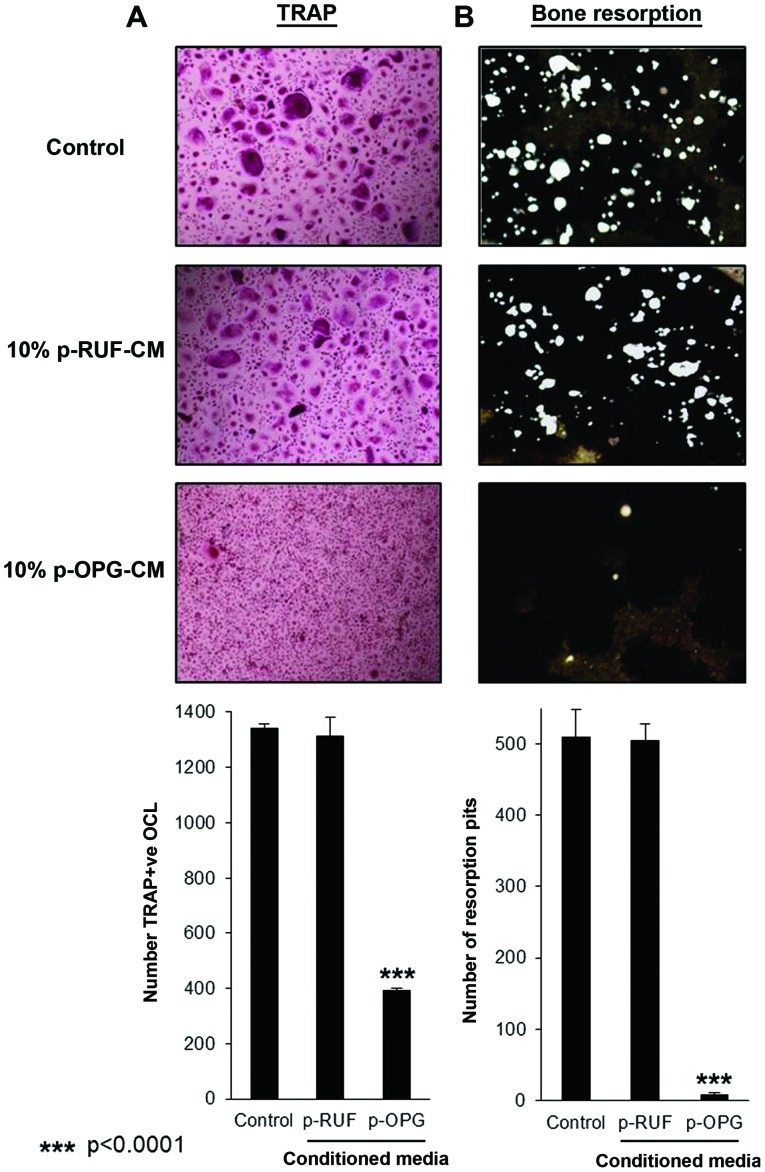
OPG produced by MDA-MB-231-TXSA cells is biologically active. (A) Human peripheral blood mononuclear cells (PBMCs) were seeded into 96-well plates and treated with 100 ng/ml of RANKL in the absence (control) or presence of 10% CM prepared from MDA-MB-231-TXSA-p-RUF or MDA-MB-231-TXSA-p-OPG cells for 7 days. Shown are representative fields of the cell cultures as indicated after TRAP staining (left panel). The number of TRAP+ve multinucleated osteoclasts (containing three or more nuclei) was scored (bottom panel). Data represent the means ± SEM of three independent experiments, p<0.0001. (B) Osteoclasts cultured from PBMCs were seeded onto plastic in 96-well plates or were directly plated onto osteologic slides in the presence or absence of CM for 7 days. Shown are representative fields of the cell cultures after Von Kossa staining (left panel). Osteologic slides were examined using a light microscope, and the number of resorption pits was counted (bottom panel). Results are shown as average number of pits (±SEM), p<0.0001.

To determine whether the OPG secreted by the transfected breast cancer cells was biologically active, we performed *in vitro* osteoclast formation assays in the presence of condition media from control and OPG overexpressing cell lines. Consistent with the role of OPG in inhibiting osteoclast differentiation and bone resorption, we found that when PBMCs were cultured with the receptor activator of nuclear factor κB ligand (RANKL), CM (10%) from p-OPG-overexpressing cells, but not from p-RUF empty vector transfected cells, dramatically inhibited the formation of TRAP^+^ multinucleated cells (Fig. 1A). Further, bone resorption by mature osteoclasts (OCL) derived from PBMCs was almost totally abolished by the transfected cell CM (Fig. 1B).

### OPG overexpressed by breast cancer cells does not affect total tumor burden in vivo

**Figure 2:**
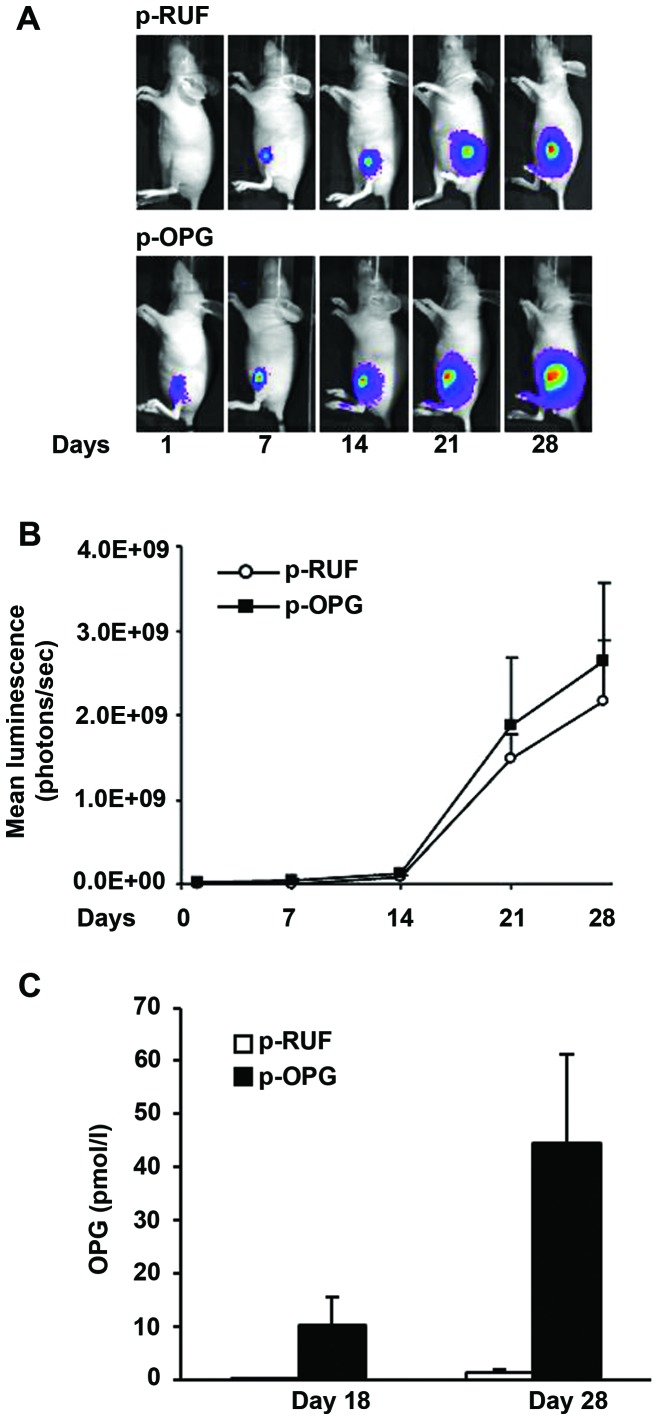
Effect of OPG overexpression by breast cancer cells on tumor growth. (A) Five week old female nude mice were injected with 0.5×10^5^ MDA-MB-231-TXSA-TGL-pRUF or p-OPG cells directly into the marrow cavity of their right tibia. Mice were imaged weekly using the Xenogen IVIS 100 bioluminescence imaging system. Representative whole body BLI images of a single animal from each group (n=10/cell line) during the course of the experiments are shown. All animals, in both groups, were humanely sacrificed on Day 28 for ethical reasons, due to high tumor load. (B) The line graph represents the average tumor signal over time measured as mean photon counts per second and it demonstrates that OPG overexpression has no effect on tumor growth when compared to p-RUF tumors. (C) OPG concentration in the blood serum of mice (n=10/cell line) collected at two different time points during the experiment as measured by ELISA, bars ± SEM.

To evaluate the effect of OPG overexpression by breast cancer cells on tumor growth we used an animal model in which the empty vector or OPG-overexpressing MDA-MB-231-TXSA breast cancer cells were transplanted directly into the tibial marrow cavity of female athymic nude mice. Bioluminescence imaging provided sensitive real-time *in vivo* assessment of breast cancer growth in bone. Transfected cells were co-transduced with a triple-fusion protein reporter construct encoding herpes simplex virus thymidine kinase (TK), green fluorescent protein (GFP) and firefly luciferase (Luc), as described previously (15,16). Development of breast cancer-induced bone destruction was assessed using high-resolution μCT analysis. Weekly imaging showed that all animals inoculated with the empty vector transfected cells showed an increase of mean photon emission, indicating an increase in tumor burden, which was clearly evident from Day 14 onwards (Fig. 2A and B). Similar results were found with mice bearing OPG-overexpressing tumors, with BLI showing no significant differences between the two groups. Bloods were collected from all animals at different time points during the experiment and the levels of circulating OPG in the mouse serum was measured by ELISA (Fig. 2C). Circulating OPG levels in animals bearing empty vector transfected cells were approximately 0.2 pmol/l, when measured on Day 18-post cancer cell transplantation. Mice bearing OPG-overexpressing tumors showed 50-fold higher serum OPG levels of 10 pmol/l in mice bearing OPG-overexpressing tumors. By Day 28, the levels of OPG in mice bearing OPG-overexpressing tumors further increased to 45 pmol/l, indicating that these cells maintained high level expression of OPG over the entire period of the study. Mice in both groups were humanely sacrificed on Day 28 due to the high tumor load in the tibiae.

### OPG overexpression inhibits cancer-induced osteolysis

**Figure 3:**
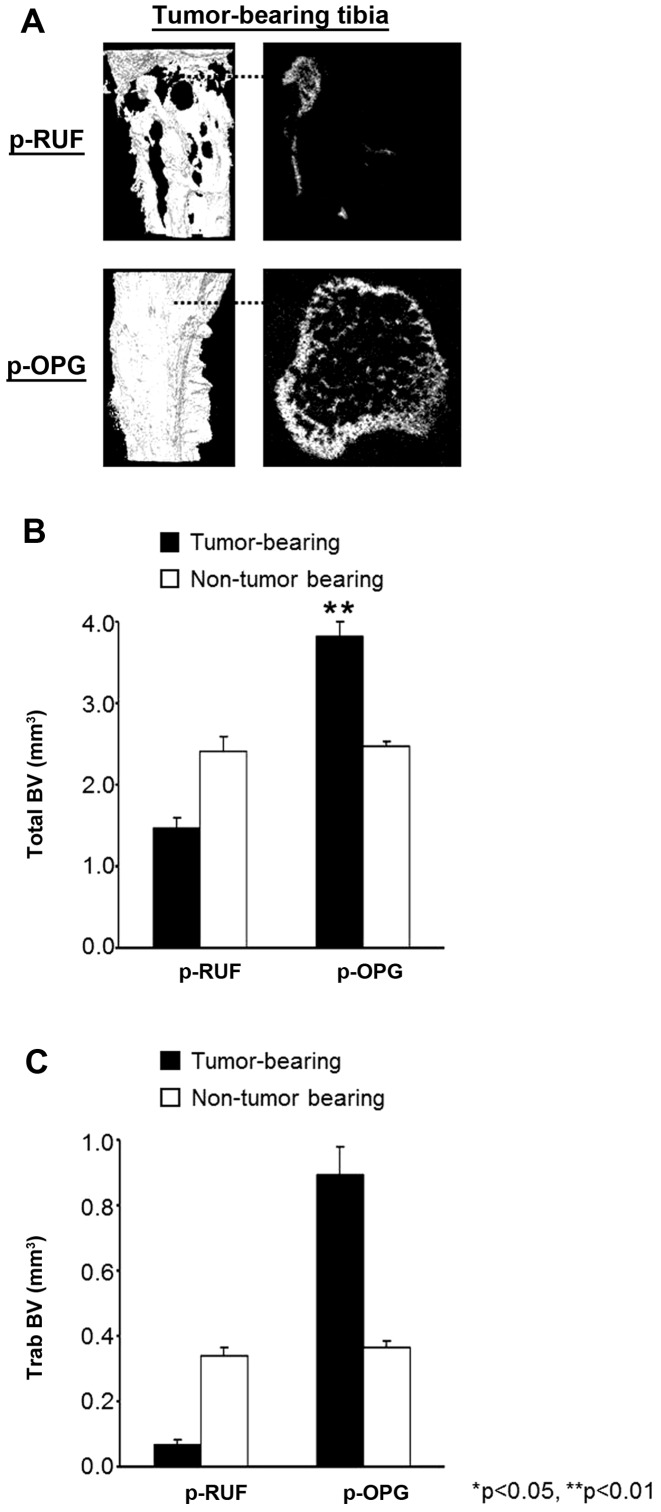
Effects of OPG overexpression by breast cancer cells on bone. (A) Qualitative 3-D m-CT images of representative animals from each group. Tibiae of mice inoculated with the empty vector transfected cells (MDA-MB-231-TXSA-pRUF) had developed large intratibial tumors and demonstrated extensive osteolysis when compared to the contralateral non-tumor bearing tibiae. In contrast, all animals inoculated with the p-OPG transfected cells showed preservation of the integrity of bone around the tumors and protection from breast cancer-induced osteolysis. (B) Quantitative assessments of total bone volume (BV) in mm^3^ in the tumor-bearing tibiae when compared to the contralateral non-tumor bearing tibiae, bars ± SEM, ^*^p<0.01. (C) Quantitative assessments of trabecular bone volume (BV) in mm^3^ when compared to the contralateral non-tumor bearing tibiae, bars ± SEM, ^*^p<0.05.

To assess the effects of OPG overexpression on bone, high-resolution μCT analysis was performed on isolated tibiae at the end of the study. Reconstructed 3-D μCT images of representative empty vector transfected tumor-bearing tibiae demonstrated extensive osteolysis when compared to the contralateral non-tumor bearing tibiae (Fig. 3A). In contrast, and consistent with the role of OPG in inhibiting osteoclastic bone resorption, all animals inoculated with the p-OPG transfected cells showed preservation of the integrity of bone around the tumors and protection from tumor-induced osteolysis, as shown in Fig. 3A. To quantify the total bone volume (BV), we compared the tumor-bearing tibiae with the contralateral non-tumor bearing tibiae of all the animals in each group in a region beginning at the growth plate and extending downwards 400×7.8 μm slices, which encompassed all of the cancer lesions. As seen in Fig. 3B, the amount of bone lost in the tibiae of mice inoculated with empty vector transfected cells exceeded 40% in the tumor-bearing tibiae when compared to the contralateral non-tumor bearing tibiae. In contrast, OPG overexpression by cancer cells resulted in remarkable protection from breast cancer-induced osteolysis, translating to a significant further increase (55%) in BV in the tibiae bearing OPG-overexpressing tumors when compared to the contralateral tibia. The effect of OPG on trabecular bone volume (TbBV) was more pronounced (Fig. 3C). Mice inoculated with the empty vector transfected cells showed a dramatic loss of their trabecular bone (>80%) when compared to the contralateral leg and to the tibiae of tumors containing OPG-overexpressing cells. In contrast, mice bearing OPG-overexpressing tumors showed a 141% gain in their TbBV when compared to the contralateral right tibiae bars ± SEM, ^*^p<0.05.

### OPG secreted by breast cancer cells maintains skeletal integrity but alters the intra- and extra-medullary tumor distribution

**Figure 4:**
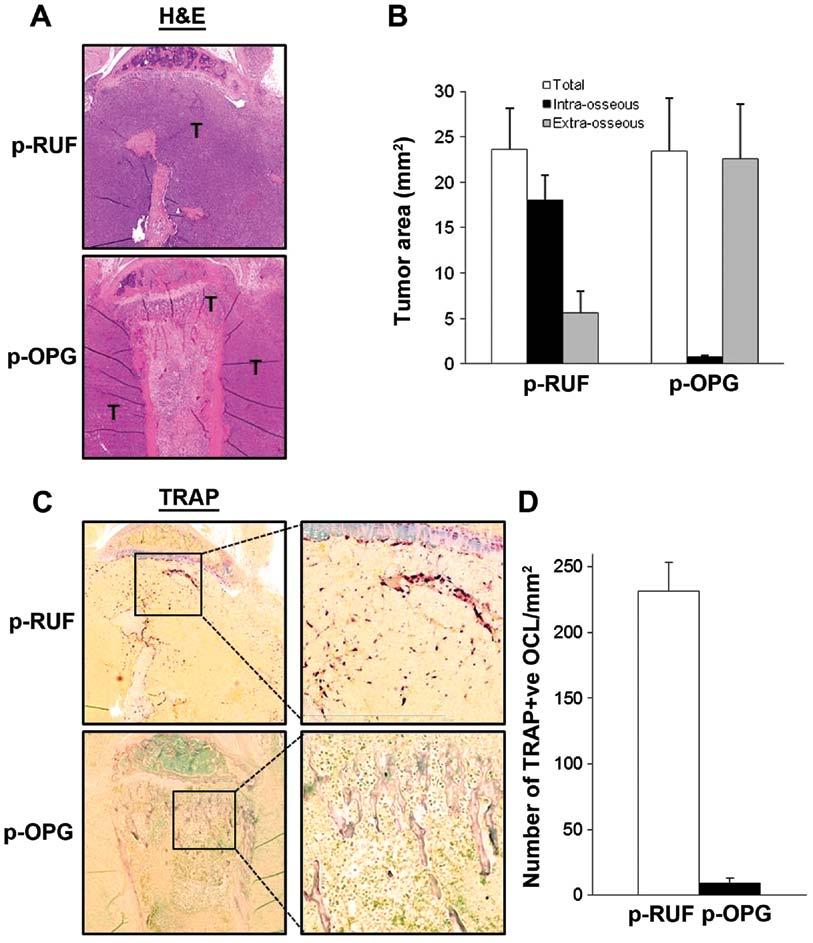
OPG overexpression maintains bone integrity but alters the intra- and extra-medullary tumor distribution. (A) Representative H&E stained tibial sections from mice inoculated with MDA-MB-231-TXSA-TGL-pRUF or p-OPG cells showing the differential distribution of intra- and extra-medullary tumor growth. (B) Quantitative assessment of intra- and extra-medullary tumor area measured in mm^2^ using the histological images. The tumor area is expressed as an average per group. (C) TRAP staining of histological sections showing absence of TRAP^+^ osteoclasts in tibiae preparations of animals inoculated with OPG transfected cells when compared to vector transfected cells in which osteoclasts were abundantly present and attached to the bone surfaces. (D) Quantitative assessment of the number of TRAP^+^ osteoclasts.

Tumor burden was evaluated using bioluminescence and showed no significant differences in the average tumor signal between the mice bearing p-OPG tumors when compared to the mice with p-RUF tumors. However, a detailed histological examination of the tibiae, using high resolution imaging, showed that the distribution of the tumor in the bone was different in animals with OPG-overexpressing tumors compared to animals bearing empty vector transfected tumors. In mice bearing empty vector transfected tumors, there was persistent growth of cancer cells within the bone marrow cavity, representing 76.4% of the overall tumor burden, which penetrated the cortical bone and invaded the surrounding soft tissue. In contrast, histological sections of tibiae with OPG-overexpressing tumors showed that the intra-osseous tumor burden was significantly decreased, accounting only for 3% of the overall tumor burden. In these tibiae, cancer cells were almost undetectable within the bone marrow space and this was also associated with a dramatic increase in trabecular bone density and a concomitant decrease in bone marrow volume (Fig. 4A). Importantly, OPG-overexpressing cancer cells escaped the marrow cavity and continued to grow in the extra-medullary space, accounting for 96.8% of the total tumor burden. Fig. 4B shows the intra and extra-osseous tumor burden in the tibiae, expressed as an average tumor area per group. The observed inhibition of osteolysis by OPG was due to the suppression of osteoclastic bone resorption since OPG released by tumor cells significantly decreased the number of osteoclasts lining the bone surface. Fig. 4C shows that TRAP^+^ osteoclasts were abundant and attached to the bone surfaces in tumor lesions from the vector only transfected cells. In contrast, there was almost complete absence of TRAP^+^ osteoclasts in tibiae from animals inoculated with p-OPG transfected cells, likely accounting for the protective effect of OPG overexpression on bone destruction and also confirming the biological activity of OPG *in vivo* (Fig. 4D).

### OPG secreted by breast cancer cells promotes pulmonary metastasis

**Figure 5:**
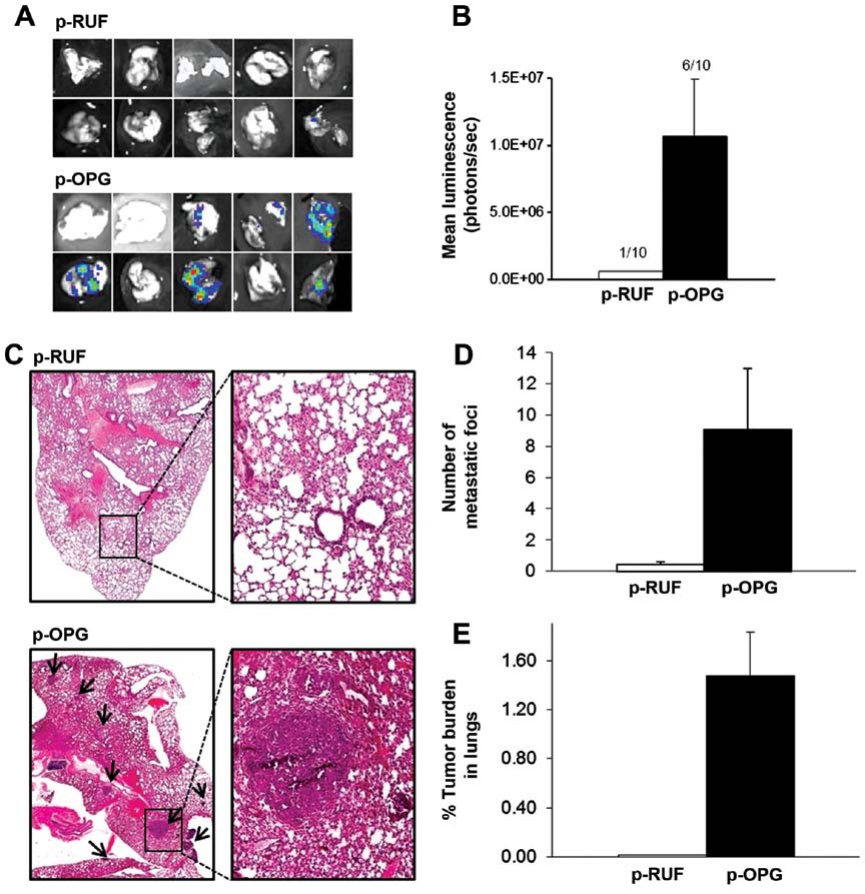
Effects of OPG overexpression on pulmonary metastasis. (A) BLI images of the lungs of all the mice inoculated with MDA-MB-231-TXSA-TGL-pRUF or p-OPG cells. From all the mice bearing tumors with empty vector transfected cells only one showed a very small BL signal whereas 6 out of 10 mice from the MDA-MB-231-TXSA-p-OPG group showed significant BL signal of pulmonary metastasis. (B) Graph represents the average metastatic BL tumor signal in the lungs measured as mean photon counts per second. (C) Representative histological sections of the lungs of mice from each group stained with H&E, confirming tumor within the lungs in the mice bearing OPG-overexpressing tumors compared to the lungs of mice bearing empty-vector transfected tumors. (D) Graph represents the average number of metastatic foci per group. Data shown in each case are an average from a representative section of each animal. Graph represents the tumor area as a percent of total and is expressed as an average per group. Bars ± SEM, ^*^p<0.01.

While OPG overexpression protected the bone from cancer-induced bone destruction, and dramatically reduced intra-osseous tumor mass, lung metastases increased significantly in OPG-overexpressing animals. At the end of the experiment the lungs from each group of animals were excised and lung metastases, as a function of photon counts per second, were quantified *ex vivo* using BLI. Six of ten animals in the OPG-overexpressing group had detectable bioluminescence signal in the lungs compared with one in ten from the group of mice inoculated with empty vector transfected cells (Fig. 5A). The mean luciferase activity in lungs from mice bearing OPG-overexpressing tumors was significantly higher than that of the animals bearing empty vector transfected tumors (Fig. 5B). Histological assessment of the lungs corroborated these findings in that multiple pulmonary macrometastases were detected in the lungs of animals with OPG expressing tumors that were also positive with BLI (Fig. 5C). The number of metastatic foci in the lungs was significantly higher in these mice when compared to the animals bearing empty vector transfected cancer cells (Fig. 5D). Tumor area was also calculated from histological sections and expressed as an average tumor area per group in absolute units (mm^2^). The percentage of tumor burden of the lung area was approximately 105-fold higher in the mice with OPG-overexpressing tumors compared to the p-RUF mice (Fig. 5E).

## Discussion

In this study we investigated the biological effect of inhibiting bone resorption and bone remodelling on the behaviour of breast cancer cells within the bone microenvironment and tested the hypothesis that inhibition of bone resorption *per se* may augment breast cancer metastasis from bone to other tissues. For this, we used a well-established xenogeneic murine model of osteolytic breast cancer, in which human breast cancer cells were transplanted directly into the tibial marrow cavity of female athymic mice. This *in vivo* model mimics the late stages of bone metastasis and is ideally suited for monitoring the effects of antiresorptive agents on breast cancer growth in the bone and also on cancer-induced bone destruction. Inhibition of bone resorption was achieved using two approaches. We engineered MDA-MB-231-TXSA breast cancer cells to overexpress native full length human OPG and, following their intratibial transplantation, tested the effects of high doses of OPG when produced and secreted locally by breast cancer cells in the bone microenvironment.

Our results show that OPG overexpression significantly reduced intra-osseous tumor burden and protected the bone from cancer-induced osteolysis. These effects were due to the actions of OPG on osteoclastic bone resorption as demonstrated by the near complete absence of TRAP^+^ osteoclasts lining the bone surface. However, despite such marked bone protection, OPG overexpression failed to reduce the total tumor burden and breast cancer cells persisted to grow unaffected in the extra-medullary space. Importantly, inhibition of bone resorption by OPG was associated with an increased tendency of breast cancer cells to metastasize to the lungs, since OPG overexpression by the breast cancer cells demonstrated a significant increase in the incidence of pulmonary metastasis, when compared to the control animals.

The data presented here are in line with our previously published data which demonstrated that inhibiting bone resorption by systemic administration of clinically relevant doses of the antiresorptive agent zoledronic acid (ZOL) had a significant protective effect on osteosarcoma (OS)-induced bone destruction but did not inhibit the total tumor burden or reduce lung metastases, and in some cases even promoted lung metastases (16). This observation was further validated by another independent study in which we showed that osteoclast inhibition by ZOL treatment increased lung metastases in mice with intra-femorally transplanted OS cells, while fulvestrant treatment lead to increases in osteoclast numbers and decreased the number of lung metastases (17). In a human setting, we conducted a transcriptomic screen of patient OS biopsies and found that expression of TRAP is significantly downregulated in primary OS compared with non-malignant bone (17). Moreover, within the OS patient cohort, patients who subsequently developed pulmonary metastasis had significantly lower TRAP expression and osteoclast numbers in their biopsies than those patients who did not develop metastasis suggesting that the metastatic potential of OS is determined in primary tumor development and that loss of osteoclasts and consequently alterations in bone remodelling in the primary lesion may enhance OS metastasis. Importantly, using the same breast cancer model as used here we have recently demonstrated that systemic administration of conventional clinical relevant doses of ZOL had no effect on tumor growth in bone but similarly promoted lung metastases in mice (18). However, metronomic doses of ZOL (i.e., lower doses given more frequently on a prolonged schedule) significantly reduced tumor burden in the tibiae of mice and reduced lung and liver metastasis when compared to the conventional treatment, suggesting that a metronomic dosing regimen may be more beneficial in the clinical setting.

Several clinical trials have shown the ability of oral and intravenous bisphosphonates to reduce the incidence and frequency of SREs and skeletal morbidity and prevent bone loss in patients with breast cancer bone metastasis when administrated alone or in combination with adjuvant therapy (5,19–24). However, results from the recent AZURE (4) trial, in which over 3000 patients with stage II/III breast cancer were randomized to receive standard therapy (chemotherapy, endocrine therapy, radiation) or standard therapy plus 4 mg of ZOL showed no significant difference between patients receiving ZOL in addition to adjuvant therapy compared to patients receiving only standard therapy, in terms of recurrence of breast cancer or on overall survival. However, a subgroup analysis that evaluated the patients receiving ZOL plus adjuvant therapy by their menopausal status, showed a significant positive effect on both recurrence and survival but only in postmenopausal women compared to patients who were pre- and peri-menopausal. Importantly, in pre- or peri-menopausal women there was an increased risk of extraskeletal metastasis. The data from the AZURE study suggest that the reproductive hormones play a role in the bone remodeling process and on the behaviour of cancer cells in the bone microenvironment, possibly contributing to cancer cell escape from bone to distant sites and implies that the hormonal environment influences the zolendronic effect on the metastatic potential of the cancer cells.

Taken together, the results of our study suggest that therapeutic agents that inhibit osteoclastic bone resorption and bone remodeling may in certain instances potentially cause more harm than good by promoting extraskeletal metastasis in patients with already established bone metastases, especially in an environment with pre-menopausal levels of reproductive hormones. These important and apparently conflicting observations suggest the need for additional research to better understand the role of osteoclast inhibition in patients with bone metastasis based on their hormonal status.
